# Association between birth characteristics and incidence of pituitary adenoma and craniopharyngioma: a registry-based study in California, 2001–2015

**DOI:** 10.1007/s10552-023-01718-7

**Published:** 2023-05-25

**Authors:** David J. Cote, Rong Wang, Libby M. Morimoto, Catherine Metayer, Gabriel Zada, Joseph L. Wiemels, Xiaomei Ma

**Affiliations:** 1 Department of Neurosurgery, Keck School of Medicine, University of Southern California, 1200 N. State Street, Suite 3300, Los Angeles, CA 90033 USA; 2 Department of Chronic Disease Epidemiology, Yale School of Public Health, New Haven, CT USA; 3 Department of Epidemiology, School of Public Health, University of California, Berkeley, CA USA; 4 Center for Genetic Epidemiology, Norris Comprehensive Cancer Center, University of Southern California, Los Angeles, CA USA

**Keywords:** Birth characteristics, Birth order, Birthweight, Craniopharyngioma, Epidemiology, Pituitary adenoma

## Abstract

**Purpose:**

To evaluate the association between birth characteristics, including parental sociodemographic characteristics, and early-onset pituitary adenoma (PA) and craniopharyngioma.

**Methods:**

Leveraging the population-based California Linkage Study of Early-onset Cancers, we identified the birth characteristics of incident cases with PA (n = 1,749) or craniopharyngioma (n = 227) who were born from 1978 to 2015 and diagnosed 1988–2015, as well as controls in a 50:1 ratio matched on birth year. Adjusted odds ratios (OR) and 95% confidence interval (CI) estimates were computed using unconditional multivariable logistic regression.

**Results:**

Males had a lower risk of PA than females (OR = 0.37, 95%CI: 0.34–0.41), and Black (OR = 1.55, 95%CI: 1.30–1.84) or Hispanic (OR = 1.53, 95%CI: 1.34–1.74) individuals had a higher risk compared to non-Hispanic Whites. Older maternal age was positively associated with PA (OR = 1.09, 95%CI: 1.04–1.15 per 5 years, p < 0.01), as was higher maternal education (OR = 1.12, 95%CI: 1.04–1.20 per year, p < 0.01). There were no statistically significant associations between birthweight (OR = 1.04, 95%CI: 0.99–1.09 per 500 g, p = 0.12), birth plurality, or birth order and PA. When stratified by race and ethnicity, the significant association with maternal education was identified only for non-Hispanic White individuals. On multivariable logistic regression, no statistically significant associations were identified between birth characteristics and incidence of craniopharyngioma, except that risk was higher among Hispanic (OR = 1.45, 95%CI: 1.01–2.08) compared to non-Hispanic White individuals.

**Conclusion:**

In this large, population-based study, female sex, older maternal age, higher maternal education, and Hispanic ethnicity and Black race compared to non-Hispanic White race, were associated with an increased risk of PA in children and young adults.

**Supplementary Information:**

The online version contains supplementary material available at 10.1007/s10552-023-01718-7.

## Introduction

Pituitary adenomas (PA) are relatively common, histologically benign lesions arising from the anterior pituitary gland, with an estimated prevalence over lifetime as high as 20% based on autopsy studies [1–4]. These tumors are commonly diagnosed in women of childbearing age due to symptoms of infertility, although overall incidence of PA is highest in those older than 40 years [2]. Craniopharyngiomas, on the other hand, are rare embryonic malformational tumors that are similarly benign histologically, but often portend a more difficult clinical course, with incidence peaking in adolescence [5–7]. Despite being histologically benign, both PA and craniopharyngioma can cause significant morbidity, including headache, permanent neurologic dysfunction, vision loss, and hypopituitarism due to mass effect on nearby structures within the finite volume of the skull [1, 8, 9].

Few studies have examined risk factors for PA or craniopharyngioma [10–13]. Demographic features, including race and ethnicity, have been shown to be associated with incidence of PA and craniopharyngioma in large series, but these studies rarely adjust for other possible risk factors [2, 14, 15]. Specifically, several studies have demonstrated higher incidence of PA and craniopharyngioma in non-White as compared to White populations, particularly when comparing Black to White individuals [2]. In addition, limited evidence suggests that early life exposures may play a role in the etiology of PA, with a possible positive association between higher birthweight and later incidence [10]. Despite these findings, few studies have examined the association between early life exposures, such as birth characteristics, and later incidence of these tumors.

Given their similar clinical presentations, and the possibility that PA and craniopharyngioma may be subject to similar sociodemographic biases in detection and diagnosis, this study aimed to leverage the population-based California Linkage Study of Early-onset Cancers (CALSEC) to examine possible associations between birth characteristics—including birth order, birthweight, and maternal and paternal sociodemographic characteristics—and incidence of PA and craniopharyngioma in children and young adults. Our aim was to conduct a hypothesis-generating investigation using a study sample with little or no participation or selection biases.

## Methods

### Data source

The methods of the CALSEC data and sample collection have been published in detail elsewhere [16–18]. Briefly, statewide information on cancer diagnosis (for the years of 1988–2015) from the California Cancer Registry (CCR) were linked to California birth records (for the years of 1978–2015) maintained by the Vital Statistics Advisory Committee of the California Department of Public Health. The reporting of both cancer diagnosis and birth is mandated by law and is considered complete at the population level. In 2001, the CCR began recording incidence of non-malignant tumors. From CALSEC, we identified cases who were born during 1978–2015 and diagnosed with first primary and incident PA or craniopharyngioma during 1988–2015, at the age of 0–37 years, as well as 50 times as many controls who were frequency matched to cases on year of birth. For each of these individuals, we extracted data from available birth records on birthweight, gestational age, mode of delivery, birth plurality, and birth order. In addition, we retrieved demographic information, including parental race and ethnicity, maternal age at delivery, maternal education, and maternal nativity. No data were extracted from hospital or other treatment records for this analysis, and no consent was required from included individuals. The study protocol was approved by the Institutional Review Boards at the California Health and Human Services Agency, University of California, Berkeley (Berkeley, CA), the University of Southern California (Los Angeles, CA), and Yale University (New Haven, CT). Data are available from the CCR and the Vital Statistics Advisory Committee of the California Department of Public Health.

### Outcomes

Diagnoses of non-malignant brain tumors were recorded in CCR beginning in 2001. All patients diagnosed with PA or craniopharyngioma in the CCR were included as cases according to the International Classification of Diseases for Oncology, Third Edition (ICD-O-3) (**Supplementary Table 1**). Patients who had other benign or malignant tumors before PA or craniopharyngioma were excluded, given that treatment for prior disease may affect risk of later PA or craniopharyngioma. Age-adjusted incidence rates were calculated from 2004 onward using SEER*STAT to identify changes in rates over time, age-adjusted to the 2000 U.S. population.

### Statistical analysis

Overall characteristics of cases and controls were displayed as frequencies, and Pearson’s χ^2^ test were used to compare baseline characteristics between cases and controls. We computed odds ratio (OR) estimates for PA and craniopharyngioma and 95% confidence intervals (CI) using unconditional multivariable logistic regression. Models included year of birth, sex, maternal race/ethnicity (non-Hispanic White, non-Hispanic Black, Hispanic, non-Hispanic Asian/Pacific Islander, other), and birth characteristics, including mode of delivery (Caesarean vs. vaginal), birth order (first, second, or third and higher), plurality (singleton vs. multiple), birthweight (grams), gestational age (weeks), maternal age (years), maternal education (years), and maternal nativity (US vs. other). All analyses were performed using SAS (version 9.4, SAS Institute) and all tests were two-sided with an α value of 0.05.

## Results

We identified 1,749 cases of PA and 227 cases of craniopharyngioma, to which we matched 87,450 and 11,350 controls, respectively (Table 1). Compared with controls, PA cases were more likely to be female (71.8% vs. 48.8%), and Hispanic (47.7% vs. 40.5%) or non-Hispanic Black (10.2% vs. 8.7%). In addition, mothers of PA cases were older at delivery, had higher education, and were more likely to be foreign-born than those of controls. For craniopharyngioma, however, distribution of birth characteristics were similar among cases and controls. Age-adjusted incidence rate of PA per 100,000 in California increased from 1.65 (95%CI: 1.48–1.83) in 2004 to 2.22 (95%CI: 2.03–2.43) in 2019, while rate of craniopharyngioma remained stable at 0.14 (95%CI: 0.09–0.20) in 2004 and 0.13 (95%CI: 0.08–0.18) in 2019.

**Table 1:** Characteristics of included cases and controls, by tumor type

		Pituitary Adenoma			Craniopharyngioma		
**Birth Characteristic**		Cases (n = 1,749)	Controls (n = 87,450)	P-value	Cases (n = 227)	Controls (n = 11,350)	P-value
**Sex**	Female	1,256 (71.8)	42,646 (48.8)	< 0.01	105 (46.3)	5,663 (49.9)	0.28
	Male	493 (28.2)	44,804 (51.2)		122 (53.7)	5,687 (50.1)	
**Mean age at diagnosis (years, range)**		21.9 (1–37)			11.6 (0–34)		
**Race/ethnicity**	Non-Hispanic White	583 (33.3)	35,408 (40.5)	< 0.01	68 (30.0)	3,622 (31.9)	0.91
	Non-Hispanic Black	179 (10.2)	7,600 (8.7)		14 (6.2)	817 (7.2)	
	Hispanic	835 (47.7)	35,438 (40.5)		116 (51.1)	5,550 (48.9)	
	Non-Hispanic Asian/Pacific Islander	129 (7.4)	7,820 (8.9)		25 (11.0)	1,193 (10.5)	
	Other	23 (1.3)	1,184 (1.4)		4 (1.8)	168 (1.5)	
**Birth weight (grams)**	250–2499	98 (5.6)	5,166 (5.9)	0.64	15 (6.6)	692 (6.1)	0.69
	2500–2999	278 (15.9)	13,331 (15.2)		35 (15.4)	1,820 (16.0)	
	3000–3499	669 (38.3)	32,522 (37.2)		93 (41.0)	4,371 (38.5)	
	3500–3999	519 (29.7)	26,451 (30.2)		57 (25.1)	3,298 (29.1)	
	4000+	185 (10.6)	9,980 (11.4)		27 (11.9)	1,169 (10.3)	
**Gestational age (weeks)**	22–36	155 (8.9)	7,863 (9.0)	0.73	24 (10.6)	1,112 (9.8)	0.77
	37–41	1,269 (72.6)	63,295 (72.4)		173 (76.2)	8,727 (76.9)	
	42–44	175 (10.0)	9,312 (10.6)		14 (6.2)	829 (7.3)	
	Unknown	150 (8.6)	6,980 (8.0)		16 (7.0)	682 (6.0)	
**Birth plurality**	Singleton	1,717 (98.2)	85,587 (97.9)	0.39	222 (97.8)	11,077 (97.6)	0.84
	Multiple	32 (1.8)	1,863 (2.1)		5 (2.2)	273 (2.4)	
**Birth order**	1st	706 (40.4)	35,559 (40.7)	0.18	80 (35.2)	4,485 (39.5)	0.37
	2nd	522 (29.8)	27,458 (31.4)		79 (34.8)	3,545 (31.2)	
	≥ 3rd	521 (29.8)	24,433 (27.9)		68 (30.0)	3,320 (29.3)	
**Mode of delivery**	Vaginal	1,399 (80.0)	69,246 (79.2)	0.41	177 (78.0)	8,585 (75.6)	0.42
	C-section	350 (20.0)	18,204 (20.8)		50 (22.0)	2,765 (24.4)	
**Maternal age (years)**	< 20	191 (10.9)	10,487 (12.0)	0.02	18 (7.9)	1,197 (10.5)	0.50
	20–24	452 (25.8)	24,561 (28.1)		51 (22.5)	2,833 (25.0)	
	25–29	538 (30.8)	26,215 (30.0)		68 (30.0)	3,167 (27.9)	
	30–34	369 (21.1)	17,800 (20.4)		59 (26.0)	2,584 (22.8)	
	≥ 35	199 (11.4)	8,387 (9.6)		31 (13.7)	1,569 (13.8)	
**Maternal education**	≤ 8 years	113 (6.5)	5,988 (6.8)	0.03	18 (7.9)	1,141 (10.1)	0.53
	9–11 years	118 (6.7)	7,387 (8.4)		29 (12.8)	1,662 (14.6)	
	12 years	255 (14.6)	12,771 (14.6)		57 (25.1)	2,587 (22.8)	
	13–15 years	180 (10.3)	8,186 (9.4)		34 (15.0)	1,830 (16.1)	
	≥ 16 years	153 (8.7)	6,618 (7.6)		44 (19.4)	1,901 (16.7)	
	Unknown	930 (53.2)	46,500 (53.2)		45 (19.8)	2,229 (19.6)	
**Mother’s place of birth**	US	1,053 (60.2)	55,990 (64.0)	< 0.01	131 (57.7)	6,118 (53.9)	0.25
	Foreign	696 (39.8)	31,460 (36.0)		96 (42.3)	5,232 (46.1)	

On multivariable logistic regression, males had a lower risk of PA than females (OR = 0.37, 95% CI: 0.34–0.41). Black (OR = 1.55, 95% CI: 1.30–1.84) or Hispanic (OR = 1.53, 95% CI: 1.34–1.74) individuals had a higher PA risk than White non-Hispanic individuals (Table 2). Higher maternal age was positively associated with risk of PA (OR = 1.09, 95% CI: 1.04–1.15 per 5 years, p < 0.01), as was maternal education (OR = 1.12, 95% CI: 1.04–1.20 per year, p < 0.01). There were no statistically significant associations between birthweight (OR = 1.04, 95% CI: 0.99–1.09 per 500 g, p = 0.12), birth plurality, or birth order and risk of PA. Findings were similar using coarser categorization of birthweight (OR = 1.04, 95%CI: 0.92–1.18 for 3000-3999 g, OR = 1.09, 95%CI: 0.90–1.31 for ≥ 4000 g compared to < 3000 g).

**Table 2:** Logistic regression model demonstrating the association between birth characteristics and risk of pituitary adenoma

				Univariable			Multivariable		
**Birth Characteristic**		Cases (%)	Controls (%)	Odds Ratio	95% Confidence Interval	P-value	Odds Ratio^a^	95% Confidence Interval	P-value
**Sex**	Female	1,256 (71.8)	42,646 (48.8)	1.00			1.00		
	Male	493 (28.2)	44,804 (51.2)	0.37	0.34–0.41	< 0.01	0.37	0.34–0.41	< 0.01
**Race/ethnicity**	Non-Hispanic White	583 (33.3)	35,408 (40.5)	1.00			1.00		
	Non-Hispanic Black	179 (10.2)	7,600 (8.7)	1.43	1.21–1.69	< 0.01	1.55	1.30–1.84	< 0.01
	Hispanic	835 (47.7)	35,438 (40.5)	1.43	1.29–1.59	< 0.01	1.53	1.34–1.74	< 0.01
	Non-Hispanic Asian/Pacific Islander	129 (7.4)	7,820 (8.9)	1.00	0.83–1.21	0.98	0.91	0.74–1.13	0.40
	Other	23 (1.3)	1,184 (1.4)	1.18	0.77–1.80	0.44	1.22	0.80–1.86	0.36
**Birth weight (grams)**	250–2499	98 (5.6)	5,166 (5.9)	0.92	0.74–1.14	0.46	0.90	0.71–1.14	0.39
	2500–2999	278 (15.9)	13,331 (15.2)	1.01	0.88–1.17	0.85	0.99	0.86–1.14	0.88
	3000–3499	669 (38.3)	32,522 (37.2)	1.00			1.00		
	3500–3999	519 (29.7)	26,451 (30.2)	0.95	0.85–1.07	0.42	1.03	0.91–1.15	0.67
	4000+	185 (10.6)	9,980 (11.4)	0.90	0.76–1.06	0.21	1.05	0.89–1.25	0.53
	Per 500 g			0.99	0.95–1.03	0.57	1.04	0.99–1.09	0.12
**Gestational age (weeks)**	22–36	155 (8.9)	7,863 (9.0)	0.98	0.83–1.16	0.84	1.06	0.88–1.27	0.54
	37–41	1,269 (72.6)	63,295 (72.4)	1.00			1.00		
	42–44	175 (10.0)	9,312 (10.6)	0.94	0.80–1.10	0.43	0.94	0.80–1.11	0.48
	Unknown	150 (8.6)	6,980 (8.0)	1.07	0.90–1.27	0.43	1.10	0.92–1.31	0.29
**Birth plurality**	Singleton	1,717 (98.2)	85,587 (97.9)	1.00			1.00		
	Multiple	32 (1.8)	1,863 (2.1)	0.86	0.60–1.22	0.39	0.89	0.61–1.29	0.54
**Birth order**	1st	706 (40.4)	35,559 (40.7)	1.00			1.00		
	2nd	522 (29.8)	27,458 (31.4)	0.96	0.85–1.07	0.46	0.91	0.81–1.03	0.12
	≥ 3rd	521 (29.8)	24,433 (27.9)	1.07	0.96–1.20	0.22	0.94	0.83–1.07	0.37
	Trend			1.02	0.98–1.05	0.35	0.97	0.93–1.01	0.18
**Mode of delivery**	Vaginal	1,399 (80.0)	69,246 (79.2)	1.00			1.00		
	C-section	350 (20.0)	18,204 (20.8)	0.95	0.85–1.07	0.41	0.96	0.85–1.08	0.52
**Year of birth**	1978–1982	435 (24.9)	21,750 (24.9)	1.00	0.88–1.14	1.00	1.08	0.92–1.27	0.35
	1983–1987	460 (26.3)	23,000 (26.3)	1.00	0.88–1.14	1.00	1.04	0.87–1.24	0.67
	1988–1992	486 (27.8)	24,300 (27.8)	1.00			1.00		
	1993–2013	368 (21.0)	18,400 (21.0)	1.00	0.87–1.15	1.00	0.96	0.83–1.10	0.55
**Maternal age (years)**	< 20	191 (10.9)	10,487 (12.0)	0.89	0.75–1.05	0.16	0.85	0.71–1.02	0.07
	20–24	452 (25.8)	24,561 (28.1)	0.90	0.79–1.02	0.09	0.87	0.76–0.99	0.03
	25–29	538 (30.8)	26,215 (30.0)	1.00			1.00		
	30–34	369 (21.1)	17,800 (20.4)	1.01	0.88–1.15	0.88	1.03	0.90–1.18	0.67
	≥ 35	199 (11.4)	8,387 (9.6)	1.16	0.98–1.36	0.08	1.17	0.99–1.39	0.06
	Per 5 years			1.07	1.03–1.12	< 0.01	1.09	1.04–1.15	< 0.01
**Maternal education**	≤ 8 years	113 (6.5)	5,988 (6.8)	0.95	0.76–1.18	0.62	0.76	0.60–0.96	0.02
	9–11 years	118 (6.7)	7,387 (8.4)	0.80	0.64-1.00	0.05	0.75	0.60–0.94	0.01
	12 years	255 (14.6)	12,771 (14.6)	1.00			1.00		
	13–15 years	180 (10.3)	8,186 (9.4)	1.10	0.91–1.34	0.33	1.14	0.94–1.39	0.19
	≥ 16 years	153 (8.7)	6,618 (7.6)	1.16	0.95–1.42	0.16	1.26	1.02–1.56	0.03
	Unknown	930 (53.2)	46,500 (53.2)	1.00	0.87–1.15	0.98	0.98	0.81–1.19	0.87
	Trend			1.04	0.98–1.10	0.16	1.12	1.04–1.20	< 0.01
**Mother’s place of birth**	US	1,053 (60.2)	55,990 (64.0)	1.00			1.00		
	Foreign	696 (39.8)	31,460 (36.0)	1.18	1.07–1.30	< 0.01	1.12	0.99–1.27	0.07

^a^All variables were mutually adjusted in the model

When stratified by race and ethnicity (Hispanic vs. non-Hispanic White), findings were similar, although the association by maternal education was identified only for non-Hispanic White individuals (OR = 1.32, 95% CI: 1.13–1.54 per year, p < 0.01) and not Hispanic individuals (OR = 1.06, 95% CI: 0.96–1.17 per year, p = 0.26) (Table 3). Results stratified by age (0–19 years vs. ≥20 years) were similar (data not shown).

**Table 3:** Logistic regression model demonstrating the association between birth characteristics and risk of pituitary adenoma, by race/ethnicity

		Non-Hispanic White					Hispanic				
**Birth Characteristic**		Cases (%)	Control (%)	Odds Ratio	95% Confidence Interval	P-value	Cases (%)	Controls (%)	Odds Ratio	95% Confidence Interval	P-value
**Sex**	Female	412 (70.7)	5,791 (48.3)	1.00			601 (72.0)	8,526 (49.3)	1.00		
	Male	171 (29.3)	6,189 (51.7)	0.38	0.32–0.46	< 0.01	234 (28.0)	8,757 (50.7)	0.37	0.32–0.44	< 0.01
**Birth weight (grams)**	250–2499	34 (5.8)	574 (4.8)	1.19	0.78–1.82	0.43	32 (3.8)	916 (5.3)	0.79	0.53–1.18	0.24
	2500–2999	69 (11.8)	1,511 (12.6)	0.93	0.70–1.24	0.61	133 (15.9)	2,631 (15.2)	1.00	0.81–1.23	0.99
	3000–3499	202 (34.6)	4,255 (35.5)	1.00			331 (39.6)	6,657 (38.5)	1.00		
	3500–3999	204 (35.0)	3,961 (33.1)	1.16	0.95–1.42	0.14	248 (29.7)	5,107 (29.5)	1.04	0.88–1.23	0.65
	4000+	74 (12.7)	1,679 (14.0)	1.10	0.83–1.45	0.51	91 (10.9)	1,972 (11.4)	1.07	0.84–1.37	0.58
	Per 500 g			1.04	0.96–1.13	0.33			1.06	0.99–1.13	0.10
**Gestational age (weeks)**	22–36	51 (8.7)	842 (7.0)	1.39	0.99–1.95	0.06	61 (7.3)	1,616 (9.4)	0.87	0.65–1.16	0.35
	37–41	418 (71.7)	8,630 (72.0)	1.00			623 (74.6)	12,649 (73.2)	1.00		
	42–44	68 (11.7)	1,534 (12.8)	0.96	0.74–1.26	0.78	77 (9.2)	1,770 (10.2)	0.85	0.67–1.09	0.21
	Unknown	46 (7.9)	974 (8.1)	1.09	0.80–1.50	0.58	74 (8.9)	1,248 (7.2)	1.19	0.92–1.53	0.19
**Birth plurality**	Singleton	573 (98.3)	11,695 (97.6)	1.00			826 (98.9)	16,962 (98.1)	1.00		
	Multiple	10 (1.7)	285 (2.4)	0.60	0.31–1.18	0.14	9 (1.1)	321 (1.9)	0.68	0.34–1.36	0.27
**Birth order**	1st	251 (43.1)	5,305 (44.3)	1.00			319 (38.2)	6,370 (36.9)	1.00		
	2nd	196 (33.6)	4,085 (34.1)	0.96	0.78–1.17	0.66	230 (27.5)	4,958 (28.7)	0.89	0.74–1.06	0.19
	≥ 3rd	136 (23.3)	2,590 (21.6)	1.03	0.82–1.30	0.81	286 (34.3)	5,955 (34.5)	0.90	0.74–1.09	0.28
	Trend			0.99	0.91–1.07	0.74			0.98	0.92–1.03	0.44
**Mode of delivery**	Vaginal	450 (77.2)	9,411 (78.6)	1.00			681 (81.6)	13,757 (79.6)	1.00		
	C-section	133 (22.8)	2,569 (21.4)	1.07	0.87–1.31	0.54	154 (18.4)	3,526 (20.4)	0.92	0.76–1.10	0.36
**Year of birth**	1978–1982	157 (26.9)	3,962 (33.1)	0.79	0.61–1.03	0.08	182 (21.8)	2,808 (16.2)	1.50	1.14–1.96	< 0.01
	1983–1987	158 (27.1)	3,492 (29.1)	0.88	0.66–1.18	0.40	209 (25.0)	3,863 (22.4)	1.20	0.90–1.59	0.22
	1988–1992	158 (27.1)	2,933 (24.5)	1.00			243 (29.1)	5,401 (31.3)	1.00		
	1993–2013	110 (18.9)	1,593 (13.3)	1.23	0.95–1.61	0.12	201 (24.1)	5,211 (30.2)	0.86	0.70–1.04	0.12
**Maternal age (years)**	< 20	31 (5.3)	938 (7.8)	0.73	0.49–1.10	0.13	124 (14.9)	2,694 (15.6)	0.92	0.71–1.17	0.49
	20–24	124 (21.3)	3,197 (26.7)	0.81	0.64–1.02	0.08	249 (29.8)	5,553 (32.1)	0.87	0.72–1.05	0.16
	25–29	201 (34.5)	3,882 (32.4)	1.00			245 (29.3)	4,854 (28.1)	1.00		
	30–34	149 (25.6)	2,762 (23.1)	0.96	0.77–1.21	0.75	145 (17.4)	2,795 (16.2)	1.06	0.86–1.32	0.59
	≥ 35	78 (13.4)	1,201 (10.0)	1.10	0.82–1.46	0.52	72 (8.6)	1,387 (8.0)	1.11	0.84–1.46	0.48
	Per 5 years			1.10	1.01–1.20	0.03			1.07	1.00-1.16	0.05
**Maternal education**	≤ 8 years	3 (0.5)	80 (0.7)	0.70	0.21–2.29	0.55	103 (12.3)	2,727 (15.8)	0.73	0.55–0.96	0.02
	9–11 years	15 (2.6)	478 (4.0)	0.72	0.41–1.29	0.27	91 (10.9)	2,660 (15.4)	0.72	0.54–0.94	0.02
	12 years	72 (12.3)	1,577 (13.2)	1.00			139 (16.6)	2,862 (16.6)	1.00		
	13–15 years	76 (13.0)	1,346 (11.2)	1.13	0.81–1.59	0.46	64 (7.7)	1,242 (7.2)	1.07	0.78–1.45	0.68
	≥ 16 years	95 (16.3)	1,199 (10.0)	1.46	1.05–2.05	0.03	25 (3.0)	495 (2.9)	0.97	0.62–1.52	0.91
	Unknown	322 (55.2)	7,300 (60.9)	1.13	0.82–1.56	0.46	413 (49.5)	7,297 (42.2)	0.88	0.65–1.19	0.40
	Trend			1.32	1.13–1.54	< 0.01			1.06	0.96–1.17	0.26
**Mother’s place of birth**	US	513 (88.0)	10,961 (91.5)	1.00			320 (38.3)	6,783 (39.2)	1.00		
	Foreign	70 (12.0)	1,019 (8.5)	1.43	1.10–1.87	< 0.01	515 (61.7)	10,500 (60.8)	1.12	0.96–1.30	0.16

All variables were mutually adjusted in the model

**Stratified by age at diagnosis (< 14 vs. ≥14 years), findings remained similar, but significantly increased risk of PA with higher maternal age and higher birthweight were identified only in the older group (**Table 4**). Racial and ethnic differences in incidence appeared somewhat different between the two age groups, but the preponderance of cases (~ 91%) were diagnosed in the older group.**

**Table 4:** Logistic regression model demonstrating the association between birth characteristics and risk of pituitary adenoma, by age at diagnosis

		0–14 years					15–37 years				
**Birth Characteristic**		Cases (%)	Control (%)	Odds Ratio	95% Confidence Interval	P-value	Cases (%)	Controls (%)	Odds Ratio	95% Confidence Interval	P-value
**Sex**	Female	109 (63.7)	4,201 (49.1)	1.00			1,147 (72.7)	38,445 (48.7)	1.00		
	Male	62 (36.3)	4,349 (50.9)	0.57	0.41–0.78	< 0.01	431 (27.3)	40,455 (51.3)	0.35	0.32–0.40	< 0.01
**Race/ethnicity**	Non-Hispanic White	54 (31.6)	2,499 (29.2)	1.00			529 (33.5)	32,909 (41.7)	1.00		
	Non-Hispanic Black	13 (7.6)	586 (6.9)	1.21	0.64–2.28	0.55	166 (10.5)	7,014 (8.9)	1.58	1.32–1.89	< 0.01
	Hispanic	95 (55.6)	4,425 (51.8)	1.49	0.99–2.25	0.05	740 (46.9)	31,013 (39.3)	1.52	1.33–1.75	< 0.01
	Non-Hispanic Asian/Pacific Islander	9 (5.3)	936 (10.9)	0.44	0.21–0.94	0.03	120 (7.6)	6,884 (8.7)	0.98	0.79–1.23	0.87
	Other	0	104 (1.2)				23 (1.5)	1,080 (1.4)	1.35	0.88–2.07	0.17
**Birth weight (grams)**	250–2499	11 (6.4)	559 (6.5)	1.00	0.48–2.09	0.99	87 (5.5)	4,607 (5.8)	0.89	0.69–1.15	0.37
	2500–2999	31 (18.1)	1,387 (16.2)	1.10	0.71–1.71	0.66	247 (15.7)	11,944 (15.1)	0.98	0.84–1.14	0.79
	3000–3499	66 (38.6)	3,162 (37.0)	1.00			603 (38.2)	29,360 (37.2)	1.00		
	3500–3999	55 (32.2)	2,574 (30.1)	1.02	0.71–1.47	0.91	464 (29.4)	23,877 (30.3)	1.03	0.91–1.16	0.68
	4000+	8 (4.7)	868 (10.2)	0.42	0.20–0.89	0.02	177 (11.2)	9,112 (11.5)	1.13	0.95–1.34	0.17
	Per 500 g			0.89	0.77–1.03	0.11			1.05	1.00-1.11	0.03
**Gestational age (weeks)**	22–36	14 (8.2)	871 (10.2)	0.77	0.41–1.43	0.41	141 (8.9)	6,992 (8.9)	1.09	0.90–1.32	0.38
	37–41	129 (75.4)	6,599 (77.2)	1.00			1,140 (72.2)	56,696 (71.9)	1.00		
	42–44	11 (6.4)	540 (6.3)	1.09	0.58–2.04	0.80	164 (10.4)	8,772 (11.1)	0.93	0.79–1.10	0.41
	Unknown	17 (9.9)	540 (6.3)	1.66	0.99–2.79	0.06	133 (8.4)	6,440 (8.2)	1.05	0.87–1.26	0.62
**Birth plurality**	Singleton	166 (97.1)	8,341 (97.6)	1.00			1,551 (98.3)	77,246 (97.9)	1.00		
	Multiple	5 (2.9)	209 (2.4)	1.02	0.38–2.73	0.96	27 (1.7)	1,654 (2.1)	0.86	0.57–1.29	0.46
**Birth order**	1st	73 (42.7)	3,287 (38.4)	1.00			633 (40.1)	32,272 (40.9)	1.00		
	2nd	47 (27.5)	2,718 (31.8)	0.75	0.51–1.10	0.15	475 (30.1)	24,740 (31.4)	0.93	0.82–1.05	0.25
	≥ 3rd	51 (29.8)	2,545 (29.8)	0.91	0.60–1.37	0.64	470 (29.8)	21,888 (27.7)	0.94	0.82–1.08	0.39
	Trend			0.98	0.85–1.13	0.80			0.97	0.93–1.01	0.15
**Mode of delivery**	Vaginal	120 (70.2)	6,532 (76.4)	1.00			1,279 (81.1)	62,714 (79.5)	1.00		
	C-section	51 (29.8)	2,018 (23.6)	1.37	0.97–1.93	0.07	299 (18.9)	16,186 (20.5)	0.92	0.81–1.05	0.20
**Year of birth**	1978–1982	0	0				435 (27.6)	21,750 (27.6)	1.09	0.93–1.29	0.29
	1983–1987	1 (0.6)	50 (0.6)	0.68	0.08–5.67	0.72	459 (29.1)	22,950 (29.1)	1.05	0.88–1.26	0.58
	1988–1992	24 (14.0)	1,200 (14.0)	1.00			462 (29.3)	23,100 (29.3)	1.00		
	1993–2013	146 (85.4)	7,300 (85.4)	1.06	0.66–1.69	0.81	222 (14.1)	11,100 (14.1)	0.96	0.81–1.14	0.65
**Maternal age (years)**	< 20	16 (9.4)	940 (11.0)	0.79	0.42–1.50	0.48	175 (11.1)	9,547 (12.1)	0.85	0.70–1.03	0.09
	20–24	30 (17.5)	2,093 (24.5)	0.65	0.40–1.04	0.07	422 (26.7)	22,468 (28.5)	0.89	0.78–1.02	0.09
	25–29	51 (29.8)	2,258 (26.4)	1.00			487 (30.9)	23,957 (30.4)	1.00		
	30–34	50 (29.2)	2,022 (23.6)	1.08	0.72–1.62	0.73	319 (20.2)	15,778 (20.0)	1.02	0.88–1.18	0.81
	≥ 35	24 (14.0)	1,237 (14.5)	0.85	0.51–1.41	0.52	175 (11.1)	7,150 (9.1)	1.22	1.02–1.47	0.03
	Per 5 years			1.04	0.89–1.22	0.60			1.10	1.04–1.16	< 0.01
**Maternal education**	≤ 8 years	13 (7.6)	1,085 (12.7)	0.60	0.31–1.17	0.13	100 (6.3)	4,903 (6.2)	0.79	0.62–1.02	0.07
	9–11 years	23 (13.5)	1,509 (17.6)	0.78	0.46–1.33	0.36	95 (6.0)	5,878 (7.4)	0.74	0.58–0.95	0.02
	12 years	42 (24.6)	2,352 (27.5)	1.00			213 (13.5)	10,419 (13.2)	1.00		
	13–15 years	39 (22.8)	1,652 (19.3)	1.33	0.85–2.09	0.21	141 (8.9)	6,534 (8.3)	1.09	0.88–1.36	0.42
	≥ 16 years	45 (26.3)	1,616 (18.9)	1.75	1.07–2.84	0.02	108 (6.8)	5,002 (6.3)	1.13	0.89–1.45	0.31
	Unknown	9 (5.3)	336 (3.9)	1.95	0.87–4.40	0.11	921 (58.4)	46,164 (58.5)	0.95	0.78–1.15	0.59
	Trend			1.30	1.10–1.53	< 0.01			1.08	1.00-1.17	0.05
**Mother’s place of birth**	US	103 (60.2)	4,655 (54.4)	1.00			950 (60.2)	51,335 (65.1)	1.00		
	Foreign	68 (39.8)	3,895 (45.6)	0.92	0.63–1.35	0.68	628 (39.8)	27,565 (34.9)	1.14	1.00-1.30	0.05

All variables were mutually adjusted in the model

**Table 5:** Logistic regression model demonstrating the association between birth characteristics and risk of craniopharyngioma

				Univariable			Multivariable		
**Birth Characteristic**		Cases (%)	Controls (%)	Odds Ratio	95% Confidence Interval	P-value	Odds Ratio^a^	95% Confidence Interval	P-value
**Sex**	Female	105 (46.3)	5,663 (49.9)	1.00			1.00		
	Male	122 (53.7)	5,687 (50.1)	1.16	0.89–1.51	0.28	1.16	0.89–1.51	0.28
**Race/ethnicity**	Non-Hispanic White	68 (30.0)	3,622 (31.9)	1.00			1.00		
	Non-Hispanic Black	14 (6.2)	817 (7.2)	0.91	0.51–1.63	0.76	0.97	0.54–1.76	0.93
	Hispanic	116 (51.1)	5,550 (48.9)	1.11	0.82–1.51	0.49	1.45	1.01–2.08	0.04
	Non-Hispanic Asian/Pacific Islander	25 (11.0)	1,193 (10.5)	1.12	0.70–1.77	0.64	1.35	0.80–2.26	0.26
	Other	4 (1.8)	168 (1.5)	1.27	0.46–3.52	0.65	1.41	0.50–3.99	0.51
**Birth weight (grams)**	250–2499	15 (6.6)	692 (6.1)	1.02	0.59–1.77	0.95	1.06	0.57–1.96	0.86
	2500–2999	35 (15.4)	1,820 (16.0)	0.90	0.61–1.34	0.61	0.93	0.62–1.39	0.72
	3000–3499	93 (41.0)	4,371 (38.5)	1.00			1.00		
	3500–3999	57 (25.1)	3,298 (29.1)	0.81	0.58–1.13	0.22	0.79	0.56–1.10	0.16
	4000+	27 (11.9)	1,169 (10.3)	1.09	0.70–1.67	0.71	1.05	0.68–1.63	0.83
	Per 500 g			1.04	0.93–1.17	0.51	1.03	0.91–1.18	0.61
**Gestational age (weeks)**	22–36	24 (10.6)	1,112 (9.8)	1.09	0.71–1.68	0.70	1.06	0.65–1.71	0.83
	37–41	173 (76.2)	8,727 (76.9)	1.00			1.00		
	42–44	14 (6.2)	829 (7.3)	0.85	0.49–1.48	0.57	0.87	0.50–1.51	0.61
	Unknown	16 (7.0)	682 (6.0)	1.18	0.71–1.99	0.52	1.19	0.70-2.00	0.52
**Birth plurality**	Singleton	222 (97.8)	11,077 (97.6)	1.00			1.00		
	Multiple	5 (2.2)	273 (2.4)	0.91	0.37–2.24	0.84	0.88	0.34–2.27	0.79
**Birth order**	1st	80 (35.2)	4,485 (39.5)	1.00			1.00		
	2nd	79 (34.8)	3,545 (31.2)	1.25	0.91–1.71	0.16	1.21	0.87–1.67	0.26
	≥ 3rd	68 (30.0)	3,320 (29.3)	1.15	0.83–1.59	0.41	1.06	0.73–1.55	0.74
	Trend			1.03	0.93–1.13	0.62	1.00	0.89–1.13	0.96
**Mode of delivery**	Vaginal	177 (78.0)	8,585 (75.6)	1.00			1.00		
	C-section	50 (22.0)	2,765 (24.4)	0.88	0.64–1.20	0.42	0.85	0.61–1.18	0.32
**Year of birth**	1978–1994	80 (35.2)	4,000 (35.2)	1.00	0.73–1.38	1.00	1.01	0.68–1.50	0.96
	1995–2001	75 (33.0)	3,750 (33.0)	1.00	0.72–1.39	1.00	0.99	0.71–1.38	0.95
	2002–2012	72 (31.7)	3,600 (31.7)	1.00			1.00		
**Maternal age (years)**	< 20	18 (7.9)	1,197 (10.5)	0.70	0.42–1.18	0.18	0.69	0.39–1.22	0.20
	20–24	51 (22.5)	2,833 (25.0)	0.84	0.58–1.21	0.35	0.82	0.56–1.20	0.31
	25–29	68 (30.0)	3,167 (27.9)	1.00			1.00		
	30–34	59 (26.0)	2,584 (22.8)	1.06	0.75–1.51	0.73	1.09	0.76–1.56	0.66
	≥ 35	31 (13.7)	1,569 (13.8)	0.92	0.60–1.41	0.70	0.94	0.60–1.48	0.80
	Per 5 years			1.04	0.94–1.16	0.46	1.03	0.90–1.18	0.67
**Maternal education**	≤ 8 years	18 (7.9)	1,141 (10.1)	0.72	0.42–1.22	0.22	0.73	0.41–1.28	0.27
	9–11 years	29 (12.8)	1,662 (14.6)	0.79	0.50–1.24	0.31	0.82	0.51–1.30	0.39
	12 years	57 (25.1)	2,587 (22.8)	1.00			1.00		
	13–15 years	34 (15.0)	1,830 (16.1)	0.84	0.55–1.29	0.44	0.84	0.54–1.29	0.42
	≥ 16 years	44 (19.4)	1,901 (16.7)	1.05	0.71–1.56	0.81	1.06	0.68–1.65	0.81
	Unknown	45 (19.8)	2,229 (19.6)	0.92	0.62–1.36	0.66	0.93	0.58–1.49	0.77
	Trend			1.02	0.91–1.15	0.72	1.04	0.89–1.22	0.61
**Mother’s place of birth**	US	131 (57.7)	6,118 (53.9)	1.00			1.00		
	Foreign	96 (42.3)	5,232 (46.1)	0.86	0.66–1.12	0.26	0.74	0.53–1.03	0.07

^a^All variables were mutually adjusted in the model

On multivariable logistic regression, no statistically significant associations were identified between birth characteristics and incidence of craniopharyngioma, except that risk was higher among Hispanic (OR = 1.45, 95% CI: 1.01–2.08) compared to White individuals (Table 4).

## Discussion

Our study was the first to examine birth characteristics and risk of early-onset PA and craniopharyngioma. Using a relatively large number of population-based cases and matched controls, we identified positive associations between Black race and Hispanic ethnicity and incidence of PA, compared to non-Hispanic White individuals. In addition, we observed a positive association between older maternal age and higher maternal education and risk of PA, without statistically significant findings identified for birthweight or other birth characteristics. Analyses for craniopharyngioma were limited by a smaller number of identified cases, and no statistically significant associations were identified by birth characteristics for these tumors except higher incidence among Hispanic compared to White individuals.

Craniopharyngiomas—especially of the adamantinomatous subtype—commonly occur in childhood and are embryologic in origin, arising from remnants of Rathke’s pouch. No clear underlying genetic susceptibilities have been identified and as a result, it is plausible that early life exposures may play a role in incidence [6]. PAs, on the other hand, have highest incidence in adults, and are much more common among women than among men. These tumors are frequently identified in women of childbearing age, given associated symptoms of amenorrhea and infertility.

The existing literature is limited in evaluation of early life risk factors for PA and craniopharyngioma, but a recent prospective cohort study identified a possible positive association between birthweight and risk of PA (hazard ratio = 1.57, 95% CI: 1.01–2.42 comparing those > 8.5 lbs. vs. <7 lbs. at birth), but primarily included individuals with PA diagnosed as adults [10]. The same study also identified a positive association between higher young adult BMI and higher incidence of PA [10]. In the current study, no significant association was identified for incidence of PA by birthweight, although a weak trend in the same direction as the previously identified association was observed (OR = 1.05, 95%CI: 0.99–1.09 per 500 g).

Several studies have also identified differences in incidence of each of these tumors by race and ethnicity. A large study of the Central Brain Tumor Registry of the United States incorporating data from 2009 to 2013 identified higher incidence of PA among Black and Hispanic individuals and significantly lower incidence among American Indian and Alaska Natives compared to White non-Hispanic individuals [2]. The same study identified highest incidence of craniopharyngioma among Black individuals compared to other groups. In general, these disparities tended to be more pronounced among older cases (> 40 years) than younger, which may be similar to the findings in our study [2]. A 2012 SEER study incorporating data from 2004 to 2008 also identified a non-significant positive association between Black compared to White race and incidence of craniopharyngioma (RR = 1.26, 95% CI: 0.98–1.59, p = 0.07) [19]. Similarly, the current study identified higher incidence of PA among Black and Hispanic individuals, but identified higher incidence of craniopharyngioma among Hispanic rather than Black individuals.

Novel findings of the current study include a statistically-significant positive association between maternal education and incidence of PA. This finding may be attributable to an underlying disparity in incidence of PA by socioeconomic status, with higher incidence among individuals of higher socioeconomic status due to better access to medical care and a higher degree of medical screening [20]. Such a disparity has been previously suggested and may be particularly pronounced for PA, given that a large proportion of these tumors are incidentally diagnosed [3, 21]. No such trend was identified for craniopharyngioma, which may be attributable to the overall lower rate of incidental diagnosis for these tumors, or because of lower statistical power. In either case, if higher risk is attributed to a higher degree of medical screening or exposure, the observed findings for racial and ethnic disparities would be opposite to typical observed medical disparities by race/ethnicity [22, 23]. In studies of other types of cancer, for example, observed rates are lower among racial and ethnic minorities, which has been attributed to worse access to care [22–24], but in this study, rates were higher among Black and Hispanic individuals. The observed racial and ethnic disparities in incidence in this study may have been underestimates of the true disparities.

Additionally, a statistically significant trend of higher incidence of PA among individuals born to older mothers was identified, primarily driven by increased risk among those with mothers ≥ 35 years old. Similar findings have been identified for other tumor types, including acute lymphoblastic leukemia and cancers of the brain [25, 26]. The underlying mechanism of this finding cannot be ascertained by these data, but possible explanations include the accrual of chromosomal aberrations and mutations in maternal oocytes with aging, the inheritance of additional epigenetic mutations, or age-related changes in hormonal levels. Given recent evidence that use of exogenous hormones, including menopausal hormone therapy and possible oral contraceptives, may be associated with PA in the individual experiencing the exposure, a hormone-related mechanism may be plausible between the exposed mother and her child [11]. Alternatively, older maternal age may be a similar socioeconomic marker as education, but additional studies aiming to identify epigenetic associations with PA incidence may also prove fruitful, given this association.

Age-stratified analyses were limited by overall lower power due to stratification. Significant findings for higher risk of PA with higher birthweight and higher maternal age were significant only among older individuals, though the majority of cases (~91%) were diagnosed in the older group.

Limitations of the current study relate primarily to the included cases. Because registration of PA incidence in CCR only began in 2001 and collection of birth characteristics began in 1978, the oldest included cases are only 37 years old. Because PAs are relatively common in young women, the overall number of accrued cases was still adequate for statistical analysis, but it is important to note that the results reported here may not be generalizable to individuals diagnosed with PA later in life. For craniopharyngioma, despite highest incidence in adolescence, we identified relatively few cases due to the overall rarity of these tumors. In addition, we were limited to ICD-O codes rather than ICD-10 codes for both tumor types, which meant that we lacked data on important tumor characteristics, such as clinical severity and functional status (for PA). Some individuals were missing data for the included birth characteristics. Lastly, we conducted numerous statistical tests in this exploratory analysis, and it remains possible that some results may be false positives. Replication of these findings in additional datasets is imperative.

Despite these limitations, this study provides a comprehensive investigation of the association between birth characteristics and incidence of PA and craniopharyngioma, harnessing data from a comprehensive registry of non-malignant brain tumor cases in the most populous and diverse state in the United States. The results demonstrate for the first time fairly strong positive associations between maternal education at birth and maternal age at birth and later incidence of PA. In addition, this study is consistent with the existing literature, which shows higher incidence of PA among Black and Hispanic individuals compared to White individuals, and among women as compared to men. Further studies should aim to replicate these findings in different datasets, and for individuals diagnosed with these tumors later in life. Additionally, larger studies aiming to determine genetic and epigenetic susceptibilities for these tumors should be pursued given observed disparities by maternal age and by race and ethnicity in early life.

## Conclusions

Birth characteristics, including Hispanic ethnicity and Black race compared to White race, higher maternal education, and higher maternal age, are associated with later incidence of PA. Findings by maternal education, which may be evidence of an underlying disparity by socioeconomic status, were identified only for White individuals. No significant associations were identified between birth characteristics and incidence of craniopharyngioma, except higher incidence among Hispanic compared to White individuals.

## Electronic Supplementary Material

Below is the link to the electronic supplementary material.

Supplementary Material 1

## Data Availability

Data are available from the CCR and the Vital Statistics Advisory Committee of the California Department of Public Health.
