# Supplementary material for: Association between birth characteristics and incidence of pituitary adenoma and craniopharyngioma: a registry-based study in California, 2001–2015
Source: Cancer Causes Control. 2023 May 25;34(9):757–68. doi: 10.1007/s10552-023-01718-7 (PMC10363066; doi:10.1007/s10552-023-01718-7)
Supplement: Supplementary file 1 — Supplementary Material 1 [file 10552_2023_1718_MOESM1_ESM.docx]

**Supplementary Table 1.** International Classification of Diseases for Oncology, third edition coding for tumors included in this analysis.

| **Type** | **Site Code** | **Histology/Behavior** |
| --- | --- | --- |
| Tumors of the pituitary | C75.1 | 8040/0,1, 8140/0,1, 8146/0, 8260/0, 8270/0, 8271/0, 8272/0, 8280/0, 8281/0, 8290/0, 8300/0, 8310/0, 8323/0, 9492/0, 9582/0 |
| Craniopharyngioma | Any | 9350/1, 9351/1, 9352/1 |
